# Human trafficking risk factors, health impacts, and opportunities for intervention in Uganda: a qualitative analysis

**DOI:** 10.1186/s41256-023-00332-z

**Published:** 2023-12-11

**Authors:** Robin E. Klabbers, Andrea Hughes, Meredith Dank, Kelli N. O’Laughlin, Mutaawe Rogers, Hanni Stoklosa

**Affiliations:** 1 Department of Emergency Medicine, University of Washington, Seattle, WA USA; 2 Department of Global Health, University of Washington, Seattle, WA USA; 3 Independent Research Consultant, New York, NY USA; 4 Marron Institute of Urban Management, New York, NY USA; 5 Uganda Youth Development Link, Kampala, Uganda; 6 Department of Emergency Medicine, Brigham and Women’s Hospital, Boston, MA USA; 7 Department of Medicine, Brigham and Women’s Hospital, Boston, MA USA; 8 HEAL Trafficking, Long Beach, CA USA

**Keywords:** Human trafficking, Forced labor, Uganda, Public health, Prevention

## Abstract

**Background:**

Human trafficking is a global public health issue that is associated with serious short- and long-term morbidity. To address and prevent human trafficking, vulnerabilities to human trafficking and forces sustaining it need to be better understood among specific subpopulations. We aimed to explore risk and protective factors for human trafficking, the health impact of exploitation, and barriers and facilitators of seeking help throughout the human trafficking trajectory among forced labor and sex trafficking victims in Kampala, Uganda.

**Methods:**

Between March and November 2020, in-depth, semi-structured qualitative interviews were conducted with 108 victims of forced labor and sex trafficking who had completed a human trafficking survey conducted by the Uganda Youth Development Link (UYDEL). Participants who experienced various forms of exploitation were purposively invited for qualitative interviews and a convenience sample was interviewed. Interviews explored personal history, trafficking recruitment, experiences of exploitation and abuse, and experiences seeking help. Interviews were analyzed using a combination of deductive and inductive thematic analysis. Themes and subthemes were organized using an adapted conceptual framework of human trafficking.

**Results:**

Poverty and an abusive home life, frequently triggered by the death of a caretaker, underpinned vulnerability to human trafficking recruitment. Limited education, lack of social support, and survival needs pushed victims into exploitative situations. Victims of human trafficking were systematically exploited and exposed to dangerous working conditions. Victims suffered from sexually transmitted diseases, incontinence, traumatic fistulae, musculoskeletal injuries, and mental health symptoms. Lack of awareness of resources, fear of negative consequences, restrictions on movement, and dependence on the trafficker and exploitation income prevented victims from seeking help. The police and healthcare workers were the few professionals that they interacted with, but these interactions were oftentimes negative experiences.

**Conclusions:**

To address and prevent human trafficking, localized interventions are needed at all stages of the human trafficking trajectory. Health impacts of human trafficking are severe. As some of the few professionals trafficking victims interact with, police and healthcare workers are important targets for anti-trafficking training. Improved understanding of human trafficking drivers and barriers and facilitators to seeking help can inform the design of necessary interventions.

**Supplementary Information:**

The online version contains supplementary material available at 10.1186/s41256-023-00332-z.

## Background

Human trafficking is defined by the United Nations Office on Drugs and Crime as the action of “*recruitment, transportation, transfer, harboring or receipt of people”* using “*force, fraud or deception”* to achieve the goal of “*exploiting them for profit”* [1]. Human trafficking encompasses sexual exploitation, forced labor, slavery, servitude, and the removal of organs [2]. Recruiting, transporting, transferring, harboring or receiving a child under the age of 18 for the purpose of exploitation is always considered trafficking, even when force, fraud, or deception is not present [2]. An estimated 27.6 million people are exploited worldwide [3].

Human trafficking has historically been regarded as a criminal justice issue and has consequently been responded to through regulatory and law enforcement action. In recent years, however, there is increasing recognition of the importance of taking a public health approach to human trafficking [4, 5]. Framing human trafficking as a global public health issue draws attention to its health impacts on victims and its preventable nature. Human trafficking victims suffer detrimental physical and mental health impacts in the short- and long-term as a result of the hazardous working conditions and spectrum of abuse that they are exposed to [6]. The first step to prevent human trafficking, and to reach Sustainable Development Target 8.7: to eradicate forced labor, modern slavery, and human trafficking by 2030, is to understand the complex interplay among risk and resilience factors.

Human trafficking has been presented by Zimmerman et al. as a series of sequential event-related stages: recruitment, travel and transit, exploitation, and integration (and in some cases re-trafficking/re-integration) [7]. Conceptualizing trafficking in this way, presenting it as a series of stages that victims may pass through, emphasizes the cumulative nature of health risks across this trajectory and highlights the different timepoints at which intervention could take place. In 2017, Zimmerman et al. published a second model that focuses on labor exploitation specifically. This model reiterated that exploitation and harm occur throughout all stages of the exploitation process, but added a focus on social and economic inequalities as structural drivers of exploitation [4]. Both models underscore the complexity of the human trafficking problem showing that it is influenced by multiple determinants which may vary across contexts and forms of exploitation.

Uganda is a source, transit, and destination country for human trafficking; 1,476 trafficked persons were identified in Uganda in 2020 [8]. The concealed nature of human trafficking and barriers to help-seeking make the identification of trafficked persons challenging, but estimates suggest that 2,057,000 children are involved in child labor and 7000–18,000 children are victims of sex trafficking in Uganda, with a lifetime sex trafficking prevalence as high as 11.9% in some Ugandan districts [9–11]. Given that school attendance in Uganda is only mandatory until the age of thirteen and the legal age of employment is sixteen years, children aged thirteen through fifteen are particularly vulnerable to human trafficking [8, 12]. Children are often trafficked from their villages to more industrialized centers such as Kampala, the capital of Uganda [13]. Without access to shelter and other resources, many of these children, an estimated 2,600 children aged seven to seventeen in Kampala alone, end up living on the street where they are vulnerable to exploitation through forced begging, selling of goods, sexual exploitation, and illicit activities [14, 15].

In this study, we aimed to qualitatively explore the lived experience of individuals exposed to forced labor and/or sex trafficking in Uganda using a public health conceptual model of human trafficking influenced by the 2011 Zimmerman et al. model to identify patterns in vulnerabilities to human trafficking. For each human trafficking stage, we strived to (1) identify risk factors and protective factors for human trafficking (2) assess the health impact of exploitation, and (3) explore barriers and facilitators of seeking help to leave exploitation. While there is a growing body of public health trafficking research and much is known on a general level about the risk factors for trafficking globally, a more nuanced understanding of the local modifiable drivers of human trafficking among specific subpopulations is necessary to guide the design of interventions to address it [5]. Using a public health framework to systematically explore these drivers for the unique context of human trafficking in Uganda is a novel generalizable approach that will yield locally relevant knowledge.

## Methods

### Study design

The *Prevalence of Forced Begging and Sex Trafficking in Kampala, Uganda* study took place in Kampala, Uganda between January 2020 and November 2020. It was funded by the Human Trafficking Institute and conducted by John Jay College of Criminal Justice of the City University of New York (CUNY) in collaboration with Uganda Youth Development Link (UYDEL), a non-governmental organization in Kampala focused on enhancing socioeconomic opportunities for disadvantaged youths. As part of this study, UYDEL field teams collected 1787 quantitative surveys and conducted 108 in-depth interviews with individuals aged ten years and older with lived experience of sex trafficking and minors under the age of 18 who were forced to beg and/or sell goods. Here, we report the findings from the analysis of the qualitative interview data. Quantitative survey findings were presented in a report to the Human Trafficking Institute and are available on UYDEL’s website [16].

### Participant sampling and recruitment

Participants were recruited from all five divisions (Nakawa, Central, Rubuga, Makindye and Kawempe) of the greater Kampala district in Kampala, Uganda. A snowball sampling approach was taken in which youth who were in contact with UYDEL who had been forced to beg/sell goods or were engaged in commercial sex were approached by service providers for participation in a quantitative survey about their experiences of exploitation. Participants were compensated Ugandan Shilling (UGX) 5000 (approximately $1.34, €1.24) upon completion of the survey and received UGX 5000 for each additional participant (up to a maximum of three) that they recruited to the study. The survey covered demographic characteristics, migration decisions and debt situations, work conditions and earning experience, workplace abuse and help-seeking behavior. From the group of survey completers, participants were recruited for qualitative interviews with an emphasis on recruiting those participants who were trafficked and had experienced severe exploitation, i.e. participants who experienced various forms of exploitation across multiple survey indicators. Determination sex trafficking and adult forced labor was made in accordance with the United Nations trafficking definition [1]. A stricter definition of child forced labor was applied, requiring elements of force, fraud, or coercion in accordance with International Labor Organization guidance [17]. Interested participants were provided with dates and times that UYDEL field staff would be on site and available to conduct qualitative interviews to eliminate the need to record participant contact information. From this purposively sampled population, a convenience sample of ~ 50–60 participants who had experienced sex trafficking and forced begging or selling of goods, respectively, who were able to speak either Luganda or English, and were available at the times interviewers were present, were interviewed.

### Data collection

Semi-structured interviews were conducted by six female UYDEL field staff fluent in Luganda and English with a background in social work (for a completed consolidated criteria for reporting qualitative research (COREQ) checklist see Additional file 1: Appendix A). Participants were asked about their personal history, trafficking recruitment, experiences of exploitation and abuse, and experiences seeking help from justice and social agencies (interview guide included in Additional file 2: Appendix B). Field teams were trained to recognize severe emotional distress and if distress indicators were present, participants were excluded from participation.

All interview participants provided consent (adults) or assent (children) prior to participation. To protect interview participants, a waiver of parental permission was obtained for children participating in the study as some had been placed into forced begging, selling of goods, or commercial sex work by their parents, and others were at risk of negative repercussions if their parents were to gain knowledge of their involvement in the commercial sex work industry. Age and literacy level appropriate consent/assent forms were read aloud to participants, and a hard or electronic copy was provided for participants to read along and keep.

Following informed consent/assent, interviews were conducted in a private room at a community-based organization or in private spaces in the communities where the participants were recruited with no one but the interviewer and interviewee present. Based on the participant’s preference, interviews were conducted in either English or Luganda and audio recorded with permission to facilitate transcription. Interviews lasted 40 min on average. Participants completing qualitative interviews were compensated UGX 5000 for their time. All individuals approached for study participation were provided a resource card with information on where to access services. Interviews were transcribed and translated into English when necessary by multilingual transcribers. Any mention of potentially identifying information by participants in the interviews was redacted and deleted from transcripts.

### Data analysis

#### Conceptual framework of human trafficking

**Fig. 1:**
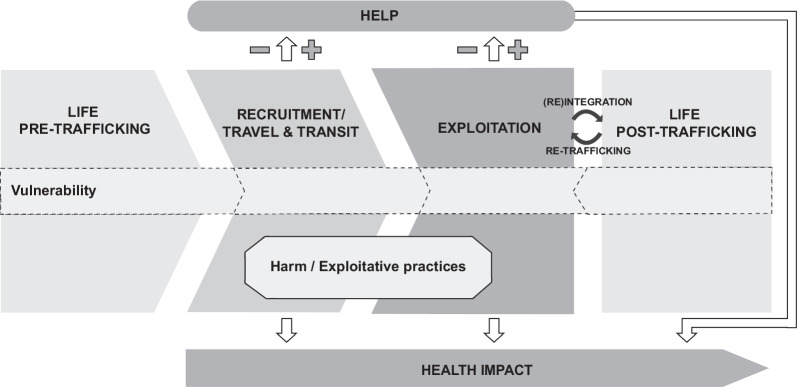
Human trafficking framework adapted from Zimmerman et al. [7]

To guide the analysis, elements from the 2011 human trafficking conceptual model and the 2017 framework of the socioeconomic determinants of labor exploitation and harm by Zimmerman et al. were combined by the research team to conceptualize human trafficking in the Ugandan context (Fig. 1) [4, 7]. The adapted conceptual framework characterized human trafficking as a multi-stage trajectory which starts with an individual’s life pre-trafficking (stage 1) in which risk and protective factors determine their vulnerability to being trafficked. At a certain point in time, individuals may be approached by an intermediary and recruited to a place of exploitation, or themselves travel to a new context where they are subsequently recruited. During this recruitment and travel/transit stage (stage 2), several risk and protective factors contribute to an individual’s vulnerability to progress to exploitation. During travel and transit, individuals may experience their first exposure to exploitative practices and harm and the associated health impacts. At this stage, barriers and facilitators influence the extent to which individuals are able to seek help. After initial recruitment, most individuals will continue to the exploitation stage (stage 3) in which they are exploited and harmed leading to negative health impacts. Various risk and protective factors influence how long individuals remain in the exploitation stage. Depending on the barriers and facilitators that are present, individuals may or may not be able to seek help during this stage to improve their circumstances. Help can take on many forms and can have an impact on the individual’s health. While for some individuals, exploitation ends and they can integrate back into society after being exploited (stage 4), many remain in exploitation or exit and are re-trafficked resulting in multiple cycles of exploitation.

Interview transcripts were split into two groups by a human trafficking expert on the research team according to whether the type of exploitation described was forced labor or sex trafficking. If both exploitation types were experienced, the focus of the interview determined to which analysis group or groups the transcript was assigned. The two groups of interviews were analyzed using separate codebooks created through a combination of deductive and inductive thematic analysis. Open coding was applied to a random sample of 10 interviews by two researchers (AH, REK) independently to identify themes and subthemes. Identified themes and subthemes were subsequently organized using the adapted conceptual framework of human trafficking designed by the research team. Organized themes were compared between the two researchers and discussed until consensus on a preliminary codebook was reached. The codebook was then applied to the remaining interviews, which were divided equally between the two researchers (random assignment of first vs second half of the list of interviews). As new themes emerged from the interviews, the codebook was iteratively refined, and after every 10–20 interviews, codes were discussed between the two researchers, resolving unclarities and discrepancies by consensus. After coding all interviews, the findings were assessed for patterns in vulnerabilities to human trafficking throughout the human trafficking trajectory. Specifically, risk and protective factors for human trafficking were considered, health impacts of exploitation, and barriers and facilitators to seeking help. For each of these, differences between the lived experience of sex trafficking and forced labor victims were noted.

## Results

### Demographic characteristics of the participants

Interviews were conducted in Kampala between March 2020 and November 2020 with a total of 108 participants. Of them, 72 participants had lived experience of sex trafficking, 31 participants had been subjected to forced labor, and 5 participants had been exposed to both types of exploitation (two analyzed as sex trafficking victims, one analyzed as a forced labor victim, and two analyzed in both groups). Most interview participants were female (80%) and the median age was 18 years (min 11, max 30 years). The interviews conducted with victims of forced labor focused on children under 18 years of age and consequently, interview participants exposed to forced labor were younger (median age 14 years versus 20 years) and had lower education levels than sex trafficking survivors (Table 1). Participants who were sex trafficking survivors were more frequently female than participants who were forced labor victims (97% vs 42%). Almost all interviews were conducted in Luganda.

**Table 1:** Participant demographics

	Interview participants		
	Forced labor (N = 31) N (%)	Sex trafficking (N = 72) N (%)	Both forms of exploitation (N = 5) N(%)
Gender			
Female	13 (42%)	70 (97%)	3 (60%)
Male	17 (55%)	2 (3%)	2 (40%)
Unknown	1 (3%)	0	0
Age			
11–13 years	16 (52%)	0	0
14–16 years	8 (26%)	8 (11%)	2 (40%)
17–19 years	6 (19%)	17 (24%)	2 (40%)
20–25 years	0	37 (51%)	1 (20%)
26–30 years	0	8 (11%)	0
Unknown	1 (3%)	0	0
Marital status			
Not married	23 (74%)	54 (75%)	5 (100%)
Married	0	5 (16%)	0
Separated	0	4 (6%)	0
Widow(er)	0	1 (1%)	0
Unknown	8 (26%)	9 (13%)	0
Highest level of schooling completed			
No schooling	6 (19%)	0	1 (20%)
Nursery	0	0	1 (20%)
Primary 1 – 4	12 (39%)	10 (14%)	2 (40%)
Primary 5 – 7	9 (29%)	41 (57%)	1 (20%)
Senior 1 – 4	0	25 (35%)	0
Unknown	3 (10%)	6 (8%)	0
Number of children			
None	21 (68%)	21 (29%)	5 (100%)
1 child	1 (3%)	26 (84%)	0
2–3 children	0	17 (24%)	0
4–5 children	0	0	0
6+ children	0	1 (1%)	0
Pregnant	0	1 (1%)	0
Unknown	9 (29%)	4 (6%)	0

**Fig. 2:**
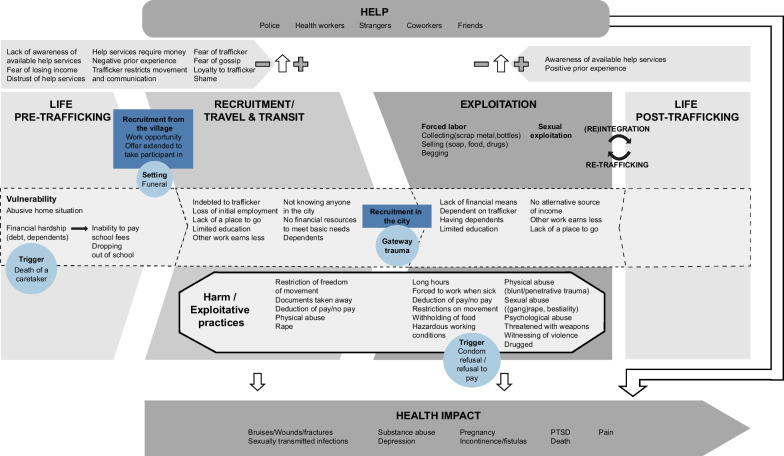
Patterns in the lived experience of forced labor and sex trafficking victims

Participants’ lived experience was explored through a public health lens. Various human trafficking risk factors, barriers to seeking help, and health impacts of exploitation and harm were identified (Fig. 2). Key findings are presented in Table 2. Similarities and differences were observed between the experiences of victims of forced labor and victims of sex trafficking. Of note, participant quotes are presented to highlight experiences, risk and protective factors, and barriers and facilitators of interest, and therefore do not always contain all the data used to determine whether participants’ experiences met criteria for human trafficking.

**Table 2:** Key interview findings by human trafficking stage

Human trafficking stage	Key findings	Illustrative interview quotes
Life circumstances pre-trafficking	• Vulnerability stems from financial hardship and abusive home situations • Vulnerability is often triggered by the death of a caretaker or the family breadwinner • In situations of financial hardship, school fees become a prohibitive cost causing children to drop out of school • Abusive home situations are often described in the context of non-blood family members (e.g., caregiver’s new partner)	*The situation that was at home was not good at all, we used to lack even just 200 UGX for sauce or just 100 UGX for food. We never used to eat, there was a time when there was no food due to the season and hence we lacked food, because you would reach the garden and in all you had planted, nothing came out.* - Sex trafficking, Female, age 20 *I didn’t take long period at school, I left school when I was 4 years as the situation at home was not good whereby there was no money, no food, to get clothes and shoes, so hard due to that situation.” Forced labor, Male, age 18* *My mother passed on, I have only my father … My stepmother was mistreating me, she used not to give me food. She could abuse me and when I told my father, he never accepted me. Instead, he could take the words of my stepmother. His wife could say, "That girl is lying! She does not want to work". So, I ran away from home.- Sex trafficking, Female, age 15*
Recruitment and travel & transit	• There is a general trend in movement from rural villages to the big city	
Recruitment in the village followed by travel and transit	• Recruitment often takes the form of an opportunity that is presented to address financial hardship (e.g., a work opportunity, or an opportunity for caregivers to relieve themselves of the financial burden of caring for a child) • Victims are recruited by family members, friends, acquaintances, and strangers Venues where recruitment takes place include funerals, sports tournaments, and dance performances • Sex trafficking: after an initial sexual trauma, victims either see no other option but to endure, or leave the exploitative situation and end up on the streets • Debt owed to the recruiter forms a barrier to leaving the exploitative situation • A minority of victims are recruited and trafficked internationally. The international context increases victims’ isolation and heightens their vulnerability	*She brought me from the village and she told me I will have a better life and there was no need to get worried. So, I also accepted to come with her. Reaching at her place, she packed bananas for me to start selling yet I didn’t want. So, I started to sell as well as being mistreated.* - Forced labor, Female, age 12 *I had a friend of mine who was working from Kampala. She would come back to the village showing us how well off she was. She told me that she was coming back to the city to get for me a job. When I reached this side, I thought she had gotten me a good job. No! It was this work of females, sex working. I also started doing that one*. - Sex trafficking, Female, age 21
Travel and transit followed by recruitment in the city	• Those arriving in the city often have no place to go and end up on the streets • Low educational attainment and lack of a social network limit access to income generating activities resulting in vulnerability to human trafficking • Sex trafficking: dependents add to the urgency of securing income for survival resulting in vulnerability to human trafficking	*My mother just took me to the bus terminal and paid the transport to the turnboy of the bus and I came alone. I wasn’t even familiar with the place because it was my first time to come this side. I meandered around Kampala town because I didn’t know where to go or what to do. I used to sleep on the streets at night.* - Forced labor, Female, age 12
Exploitation	Forced labor: • Victims are forced to beg, steal, or collect items for their exploiter to sell • Victims are made to work long days and are given little time off • Exploitation often occurs by the roadside exposing victims to the risk of road traffic accidents and environmental hazards • Victims mainly suffer physical abuse from strangers, customers, and employers and psychosocial abuse though less frequently sexual abuse also occurs • Exploiters/traffickers are often family members on whom victims are highly dependent contributing to ongoing vulnerability Sex trafficking: • Victims are often exploited in commercial sex at a bar or lodge • Victims are exposed to physical abuse from customers and employers. Refusal of sex without a condom and disputes over the price of services are common triggers of violence • Victims are exposed to (gang) rape resulting in urogenital injuries, pregnancy and sexually transmitted infections. A minority of victims report bestiality • Victims are frequently threatened and verbally abused • Mental health impacts include flashbacks, intrusive thoughts, depressive symptoms and suicidal ideations and several victims report substance abuse • Lack of financial means contributes to ongoing vulnerability. Victims require the income from exploitation for their own daily survival and that of their dependents. Other work is usually associated with lower compensation or requires start capital Barriers and facilitators of seeking help: • Barriers to seeking help during exploitation include lack of awareness of organizations that can offer help, negative experiences including not being believed and suffering further abuse in interactions with help services, fear, restrictions on movement and communication, and loyalty to and dependency on the trafficker • Facilitators of help-seeking include awareness of organizations that can offer help, positive prior experiences with seeking help, and having a support network	*There was a day when I had refused to sell bananas, she told me that, "You girl, what is making you stubborn? Yet l am not the one who gave birth to you." She locked me inside the house, beat me while strangling my neck. I was pushed on the floor till when I felt so weak, my head was hit and I felt mentally ill. I wanted report her but I feared her. Instead I just kept quiet and I have to act voiceless.* - Forced labor, Female, age 12 *They told me to sit so that put my hands forward so that people can give me money. By then I was young, but it was so embarrassing. However much I told my aunties that I didn’t like, they never listened.* - Forced labor and sex trafficking, Female, age 17 *A client can pay a boss, and the boss is the one to give that client instructions that if she refuses do this and this eee…! When I would refuse at times, a boss would tell a client that if she has refused, get hold of her by force and then sleep with her*. - Sex trafficking, Female, age 22

Warning: results section contains potentially disturbing content including graphic descriptions of violence, child abuse, sexual assault, and bestiality, which may be emotionally challenging to engage with*.* Reader discretion is advised.

### Vulnerability resulting from life-circumstances pre-trafficking

Broadly, vulnerability could be split into two main categories, namely vulnerability resulting from financial hardship and vulnerability stemming from an abusive home situation. These vulnerabilities could co-occur. Participants described living in difficult circumstances in which there was insufficient money to meet basic survival needs including food, clothing, and medicine. Often, multiple family members were dependent on a single income. The need to repay a debt caused by medical costs, funeral costs or gambling addiction compounded financial hardship in some cases. One participant described, “*My mother has some debts in the village, however her debts are ever unending, school fees debts for my young siblings and also before the death of my father he left so many debts”* (Forced labor, Female, age 11).

Financial difficulties repeatedly had a clearly identifiable trigger – the death of (one of the) the family breadwinner(s), usually a parent. Approximately half of all interview participants reported the death of a family member or caretaker. Causes of death varied and included disease, war, natural disasters, and suicide.

From primary one up to primary four, life was very easy because my dad was still there. I used to stay with my dad and my mum, and that is why life was very easy, but since my dad passed away, life became very hard. From primary five to primary six, up to primary seven, it was very hard because I was the one who was paying my [school] fees, struggling for it, and my mum didn’t have enough money. – Sex trafficking, Female, age 22

In situations of financial hardship, school fees were often an expense that could no longer be covered causing participants to drop out of school which explained the very limited education among interview participants. One participant regretted the consequences of her family’s economic circumstances for her schooling saying, *“I stopped in primary six truthfully, because of school fees issues…I liked studying, but did not have the chance”* (Sex trafficking, Female, age 18). In these dire circumstances of poverty, any work opportunity or offer to take the responsibility for a child or a child’s school fees off a parent’s hands, was considered very welcome. A participant described how her mother was presented with a financial opportunity saying, *“So when she [participant’s mother] failed to get money, there was a friend of hers who told her that there was someone who wanted a maid this side of [medium size urban neighborhood in southern Kampala], so I came. But my mother was told that I was going to be a house girl, so I came thinking that too”* (Sex trafficking, Female, age 17).

The second main determinant of vulnerability that could be identified in the interviews was an abusive home life. Interview participants gave accounts of beatings, neglect, mistreatment, withholding of food, sexual abuse, and being forced to carry out all the housework. Often, the abusive situation started when there was a new influence in the family, someone who was not a blood relative. One participant recounted how the abuse started when she moved in with her stepmother after her mother passed away:

My mother got sick and they took her to the hospital … When she died, my father took me to his mistress in [medium size urban neighborhood in Northern Kampala], that’s where we were staying, but she would mistreat me and I didn’t have any peace. … She would cook food and only give to her children, yet I was the one who would do all the work. – Sex trafficking, Female, age 18

My father gives a lot of attention to my stepmother. When she tells him something we had not done, he beats us, ties us on ropes, he starves us and [makes us] work all day. She doesn’t want to give us what to eat [something to eat]. When my father comes back, she tells him that we don’t want to work. There was a day I washed clothes. I washed three basins full of clothes. These big ones while she was beating me with a mingling stick [kitchen utensil] in the back. My back is paining! She could abuse my siblings telling them things like "Was I the one who killed your mother? You children don’t work!" While she treated her children like princesses, I even don’t know. They could also abuse us like that. – Forced labor, Male, age 15

Participants who did not feel safe or well-treated at home were often driven to run away and find alternative living arrangements. One participant described how he decided to leave the village and try his luck elsewhere saying, *“I felt tired of the village… because in the village, it was pain and mistreatment. I was tired of being tortured and beaten in the village like a thief. I got fed up and decided to go to the city.”* (Sex trafficking, Male, age 17).

The life-circumstances pre-trafficking that culminated in vulnerability to human trafficking were similar for participants who experienced sex trafficking and participants involved in forced labor.

### Risk factors for recruitment and travel & transit

Among the interviewees, there was a general trend of movement from the village where participants grew up to the “big city,” Kampala. For some participants, recruitment into exploitation took place while they were still living in the village. Other participants traveled to the city first and were recruited from there. Participants were recruited by family members, friends, acquaintances and sometimes strangers.

#### Recruitment in the village

For participants who were trafficked from the village, an “opportunity” was often presented by traffickers that leveraged the vulnerability caused by financial hardship or an abusive home situation. For those who entered forced labor, it was often a family member or an acquaintance that offered to take participants in, cover their school fees, or provide them with work. In some interviews, the venue for recruitment was at the funeral of one of the participant’s caretakers at a time when it was unclear who would support the child. Others were approached during sports tournaments and dance performances.

When my father passed on … after the burial, there was a lady who was too, too close friend to my mother. She spent mourning for one or two weeks when she was around. She said she can help me get a job, telling my mother, because my mother had no capacity to take care of us. My siblings were so many yet I was the elder, so she said I should go and work. – Sex trafficking, Female, age 18

Another participant recalled how acquaintances had offered to take her in and cover her school fees after her father passed away saying, “*During father’s burial, there came his friends saying, ‘Am going to educate such a child. Give me this child'”* (Sex trafficking, Female, age 16). The situation participants entered into, however, was rarely as promised. Instead of being sent to school, the children were put to work begging, selling, and collecting scrap. One participant described how her grandmother was deceived saying, *“she said she would take me to school, so my grandmother accepted … because she didn't have money for my school fees. She just told me to go with her. When I reached there, it was only after one week and then she started giving me pancakes to sell”* (Forced labor, sex unknown, age 11). Another participant spoke of false promises made by her uncle:

[I was] 5 years old when my uncle got me from the village and brought me here to Kampala in [small size urban location in the central business district of Kampala]. He told my parents that he was taking me to a certain school in Kampala that was offering full bursaries [scholarships] … My parents accepted because I was studying from the village and it was still hard for my parents to get school fees … I was so happy for the opportunity that I was offered, but to my surprise when I came to Kampala, things changed because after two days of my staying with them, the following day aunt decided to take me to the street so that we can beg together. I asked them, "What about the bursaries you told my father about?". My uncle told me not to question him but rather do as they say. – Forced labor and sex trafficking, Female, age 21

For participants that ended up in sex trafficking, the opportunity presented was usually a work opportunity in the city such as work in a house, hotel, or salon. Sometimes it was work that was already being done by a friend who appeared to be doing well. In many cases, the work opportunity turned out to be fictitious and what participants were actually being recruited for was commercial sex. One participant recalled how her friend visited and told her about her exciting life in Kampala saying,

One holiday, she came back to the village and told me that Kampala was so interesting. …I had a tough mother. One day, she [my mother] beat me and my heart ran to [I thought of] my friend. I said, "Why don’t I pick my things and I go?". When we came to the city, she [my friend] never told me anything. … When we arrived at [medium size urban neighborhood near the central business district of Kampala], she took me into a house but the house had only girls; I later discovered that the house was like a lodge. Men come in and use you and give you some money around five to ten thousand shillings. – Sex trafficking, Female, age 21

Once they started their new work opportunity, several participants were subjected to forced sex for the first time. These participants described a distinct initial trauma, typically rape by a customer or (a family member of) their employer. After going through this, they either chose to endure in their employment because they felt like there was no other option, or they left the work opportunity and found themselves living on the streets. After this initial gateway experience many resorted to engaging in commercial sex, and ultimately experienced sex trafficking.

When I was brought here, I was taken to her [the recruiter, a family friend’s] home. I studied for some little time while coming from her home. She was married and her husband raped me, so I left home. I met a friend who brought me here. – Sex trafficking, Female, age 23

Sometimes, participants were indebted to the trafficker who had recruited them from the village, making it difficult to leave their current situation. One participant recounted how she was not able to leave when she found out what she would be doing and said,

Yes, and that lady gave me a room and told me that I was going to start working from there, but I told her that I won’t be able to do it. But she said that I would work whether I liked it or not and that I didn’t know anyone in Kampala and that they also had to do that in order to survive. But I told her that I won’t manage and she assured me that I would manage. So when we reached, after two days, men started coming to me. The first one asked me, "Do you know this?" So, I asked him what he meant, but he just told me, "Set-up yourself quickly, I have other things to do and I have somewhere I am going". So, I also didn’t have a choice. So after five days had passed, I was getting used to it. I had nothing to do, nowhere to go, but in my mind I wanted to run. But I didn’t have a phone maybe to call my mother, had nothing on me that could help me go back home, so I just let it be. After two months I was used to everything. – Sex trafficking, Female, age 16

#### Recruitment in the city

The second group of interview participants reported coming to the city of their own volition, being abandoned in the city, or being put on a bus to the city by family members. Once on the streets, participants needed to find a means to survive. The lack of a place to go, not knowing anyone in the city, and lack of income put participants at risk of human trafficking. The limited educational background that most participants had left few options for a stable income. Many younger participants described finding safety in numbers by joining groups of street kids for survival. They then typically started doing the work that other street kids did such as collecting scrap metal, bottles, or selling soap. One participant shared his experience of first arriving in Kampala saying,

I stayed there in the city and started hustling. While I was there, I found people speaking our mother tongue. I joined them. They were home people, they understood me and I clung on them. They took me to their place. When we reached there, they told me that it was hustling throughout to sustain you. They told me that their bosses needed money therefore you had to go and look for it. I went into scrap; they took me to trenches, they have given me drugs to sell. There are those people we sell to. That is how I started living with them. – Forced labor, Male, age 15

For interview participants with lived experience of sex trafficking, who were typically older than the forced labor participants, having dependents, often children, added to the pressure to find work by any means. It was difficult to meet their own survival needs as well as those of their dependents, and they began engaging in commercial sex acts to provide for the family unit, which ultimately became a situation of sex trafficking.

I had two children! I have no job! I commenced from where I had ended. I had to look for money for both children. I could not even imagine the number of men I had to sleep with to get what to eat and yet I also had to look for money for rent. – Sex trafficking, Female, age 30

Sometimes a bad situation can drag you into something that you would not wish to do. ... First of all, I have to buy myself food, secondly I need rent, thirdly I needed school fees to educate my child. – Sex trafficking, Female, age 27

#### Human trafficking internationally

A minority of interview participants reported being trafficked internationally including to Malaysia and Dubai. One male participant described how a friend presented him with a work opportunity abroad that would allow him to pay back a debt he owed the friend,

[The friend] promised to take me to a money source so that we can work and earn. …The country he took me to was Malaysia. We arrived in a big house. We found some children, men, and girls going through torture and I asked my friend, "Is this the work you have brought me to do?" He said, "Don’t worry, this is where we get the money, and you yourself are the money." … Some of the boys would rape fellow boys. The girls were also raped. They would set cameras to record what we do and then take the recordings for sale to get money. – Sex trafficking, Male, age 19

The international setting compounded participants’ vulnerability to exploitation. Participants found themselves in a foreign country, often with no means of contacting their family, and frequently had their travel documents confiscated by the trafficker. One participant described, “*I was taken to a foreign country without my family knowing, and the contacts I had were just written on a paper. But after reaching Malaysia, they took everything we had. So, I had no way I could contact my family even if I got a chance of contacting them”* (Sex trafficking and forced labor, Female, age 21).

### Exploitation and its impacts on health

Once recruited, participants entered into a life of exploitation, which resulted in impacts on their health.

#### Forced labor exploitation

Participants who were exploited through forced labor described being made to collect different items such as scrap metal, bottles, and boxes from the street for their exploiter to sell. Others were forced to beg or steal. Participants often had to work long days, six or seven days a week, and were given almost no time off. One participant reported having no reprieve saying, *“There is no resting day unless when there is scarcity of maize [if all the maize has been sold or if it has not been brought to the market yet]. That’s when we rest. However, if it’s seen on market, we go back to work.”* (Forced labor, Male, age 14).

[I start work] at 06:00 or 05:00 in the morning....I work until 20:00, that's when I come back and of course I am always feeling tired, yet I actually have to figure out what to eat.....from the time I come back at 20:00, I eat well with what I have gotten from [medium size urban market in the central business district of Kampala] and then I go to beg ... I beg until midnight and then I come back ...I work six days in a week. – Forced labor, Female, age 17

The work frequently took place by the roadside exposing participants to the risk of road traffic accidents and environmental hazards.

Apart from that big accident, I got there some minor accidents. I get like a motorcycle knocking me, but causing minor injuries … Kampala Capital City Authority always chases us and we pass through big trenches where sewage passes, but remember we don't wear shoes because we don’t have them. – Forced labor, Male, age 13

Participants reported being harmed while carrying out their work. Most of the abuse forced labor victims were exposed to was physical. Participants described beatings from strangers, groups of older kids on the street, from customers that they sold their goods to, and from their trafficker/employers. Abuse that was mentioned included beatings with electrical cords, pouring of boiling water over them, being hit with heavy canes, being strangled, and being kicked. Psychological abuse also occurred, and participants reported being subjected to verbal abuse including insults and shaming, being threatened with beatings, not being allowed to leave without permission, and sometimes having food withheld as punishment.

Yes she forces me, when I don’t want to work. … she drags me and starts beating me severely and then she tells me to go and work. So when I come back at night, she tells me to do the housework while beating me and sometimes she ends up not giving me food. … She tells me to treat her well that she’s my mother and father...she ties me up and starts beating me and sometimes she undresses me which shames me in the presence of people. She brings my history that I am an orphan and how she got me from the rubbish pit. Then she strangles me. For sure, this situation treats me so bad and I hate myself. – Forced labor, Female, age 11

While most abuse among forced labor victims was physical, sexual abuse also occurred. Young age contributed to the vulnerability of forced labor victims. They were often dependent on others for their housing or dependent on older street children for safety while living on the street. Multiple participants provided accounts of rape and sometimes sexual exploitation by these individuals that they depended on.

So I decided to keep in the traffic jam selling such that by the time Uncle comes back he does not harass me. … I could come back home at around 10:00pm in the night and immediately he asks money from me and accompanying them with utterances like "You are coming from men". There, he forces you into the sexual acts telling me that "If you got no money, do not waste me, come over here or else I will chase you out of my home" and yet I had nowhere to go. There I bear with and I give in to him. – Forced labor, Female, age 16

For many forced labor victims, the trafficker was a family member. Victims lived with this family member and were dependent on them for food, housing and other essentials. They typically had to hand over all the money they earned and were extremely isolated.

#### Health impacts of forced labor

The abuse and dangerous exposures victims of forced labor faced in their work resulted in physical injuries that sometimes necessitated hospital admission. Reasons for consulting a healthcare professional described by forced labor victims included road traffic accidents, stepping on broken glass, and injuries from blunt and penetrating force trauma inflicted by others. The impact of their experiences on mental health was also significant and participants described how their life burdened them, how they felt sad and hated themselves.

#### Sexual exploitation

Interview participants with lived experience of sex trafficking described sexual exploitation with varying degrees of control by the exploiter. The most common situation described was that of commercial sex at a bar or lodge where the exploiter determined which customers participants saw and which types of services they provided them. Customers were seen on the lodge or bar property. Sex trafficking victims were exposed to physical, sexual, and psychological abuse during their work. Physical abuse was perpetrated by customers and employers. Participants described beatings, strangulation, being hit with a belt, cable, or brick, and being stabbed.

I have ever been hit with a bottle; customer came and we did what we had to do. Then he told me that he had no other money yet he wanted another round. So, I told him I was leaving and going somewhere else. When I was leaving, he hit me with a bottle....I don’t really know, that’s when I stopped understanding. – Sex trafficking, Female, age 16

Yes, she [exploiter] cut me with a knife here. I had refused to do what she wanted me to do. She had told me to go and sleep with a man and get money because the man had promised her 50,000 UGX so she told me to sleep with him. I said "No, I will not. What if the man is already infected with a disease and I die?". She said to me, "Haven’t you heard what I told you?". She instead beat me, got a knife which was on a table that she used it to cut me. She told me to go inside the house and stay there, I will not be given food, so I went. – Sex trafficking, Female, age 15

Common triggers for violence towards victims included participant refusal of sex without a condom and disputes over the price of services. There were many accounts of customers refusing condoms, or removing the condom before sex.

When I told him about wearing a condom, he pulled out a knife and put it on me. When he put the knife on me, I plead for mercy. I pleaded with him to have mercy on me. He had really put the knife and told me that I didn’t know the person I was playing with. "I can kill you and leave you here dead and then move away. Where will you find me?" He ignored my plea and still fucked me without putting on a condom. – Sex trafficking, Female, age 27

We always feared contracting it [human immunodeficiency virus (HIV)], but as you know men of nowadays, they come with condoms but leave them at the table. You try to quarrel a bit but they are strong and would tell your boss. We were at the mercies of God. – Sex trafficking, Female, age 21

Initiation into sex trafficking frequently happened through rape, sometimes by multiple people, and in some cases was preceded by drugging the participant. One female explained,

When they took me to a room, they used me. The man used me. After him using me, he was using my anus. When I was sobering up, I felt myself weak. I did not understand what was going on. When I started gaining my sight, I was seeing three men. I did not understand. They were big. I was nude. They had gotten their phone and were taking pictures of me while laughing. I felt my anus paining a lot. I think they took when I was not sober. When I gained my consciousness, I understood that these men had sex with me through my anus. They got out. – Sex trafficking, Female, age 23

Vaginal, anal, and oral rape occurred. In a number of interviews, participants described being subjected to bestiality with dogs and horses.

He brought his two men and caught both my legs and hands. Then he put his dog on me. That day it was not easy because even the dog scratched me in the ear at the beginning. … He then told me that, "It is you who caused it to do this, but if you come when you are calm, it doesn’t hurt in any way". I also started to learn the behaviour of the dog… They could dress it on a condom. He did not want his dog to get sick. – Sex trafficking, Female, age 16

In the morning, girls would be taken to kennels to sleep with dogs; the dogs would have sex with them. The medicine that was injected in us would make the girls seductive to themselves and they would be put to the kennels and let the dogs do all they want to them. The girls would cry, but they had no way out because they would be tied. The medicine injected in boys would enlarge our penises and they would send us to the horses. The horses also seemed to be used to this kind of treatment. You would have to have sex with the horse. – Sex trafficking, Male, age 19

Participants were frequently verbally abused and threatened verbally or with weapons. Their movement was often limited by their exploiter and sometimes food was withheld. Participants had to work when they did not want to and sometimes had to work even when sick. Money was paid either directly to the exploiter who later gave a small proportion to the participant, or participants received money from the customers directly but had to give most of it to their exploiter. It was a common occurrence that customers would not pay after sex or paid less than they had promised. When participants received money from their exploiters, sometimes unexpected fees were deducted such as the cost of the room they worked from or the cost of their meals at the bar.

#### Health impacts of sex trafficking

The health impacts of sex trafficking were significant. Health impacts could be grouped into three main categories, namely urogenital injuries (i.e., injuries of the urinary or genital organs), musculoskeletal injuries (i.e., injuries relating to bones, muscles, and soft tissues) and mental health consequences (i.e., disturbance in mood, cognition, and the ability to interact with others). Through their work, participants were at high risk of acquiring sexually transmitted infections (STIs) including HIV. One participant described the physical discomfort she felt saying, *“I got sick; I first got these diseases that catch in the private parts. It would be hard for me to walk. I would feel pain. When I go to urinate, the urine wouldn’t come out properly, it would be hurting every time I urinated”* (Sex trafficking, Female, age 18). Another participant reported contracting multiple STIs saying, *“First of all, I contracted HIV/AIDS (acquired immunodeficiency syndrome). We get syphilis infections, candida and those others illnesses that is the second. The third illness that I see coming, if I don't fight for myself, is death.”* (Sex trafficking, Female, age 21).

Rape and rough sex caused vaginal and anal injuries leading to the development of fistulas and incontinence problems. Participants described abdominal pain and localized genital pain impacting their ability to sit and to walk. One participant reported fecal incontinence saying, *“I did not have brakes behind. Wherever I sat, stools would just flow out. I was treated badly. I could not even sit at times. I would feel a lot of heat at my bottom and pain.”* (Sex trafficking, Male, age 17).

Multiple interview participants reported becoming pregnant through their work. Some participants kept the pregnancy and others sought an abortion.

I have only one child, she is 7 months old, but I was not ready to have a child because I was working on the streets and I got pregnant because I did not have safe sex … Yes, I was looking for clients and that is how I got pregnant. I could have aborted it, but I did not have money for abortion. And the little money that I had, I was using it for rent. – Sex trafficking, Female, age 20

Blunt and penetrating trauma inflicted by customers or exploiters resulted in bruises, wounds and fractures that sometimes required hospital care. A participant listed the different places where she had been wounded saying, *“Some of us have scars. Like me, I have scars at my back. They cut me with a blade. I have scars in my face”* (Sex trafficking, Female, age 20).

The experiences participants went through in their exploitation impacted their mental health. Multiple participants reported taking drugs or alcohol to be able to endure and described how others in their line of work had developed addiction. One participant explained how he used substances as a coping mechanism, saying *“You still get some problems. Nowadays, if I am going to do it [sex work], I first sniff some fuel or take drugs to relieve the pain. They do it while I am high”* (Sex trafficking, Male, age 17).

Some participants suffered from flashbacks and intrusive thoughts and displayed depressive symptoms. A minority of participants described times when they had suicidal thoughts and ideations. They told of feeling worthless.

Sometimes I pretend around so that they can pass and forget them, but am hurt inside. However much you see me eating and smiling, for sure I really don’t mean it. My heart is filled with something more than sorrow...You don’t know the sadness we have faced on this Earth! You don’t know what it means to sleep with a man you have just met on the street and then you have sex with him without you having feelings for him. – Sex trafficking, Female, age 30

#### Ongoing vulnerability

Lack of financial means was one of main factors contributing to ongoing vulnerability for both victims of sex trafficking and forced labor. While sex trafficking victims earned money through their work, after rent, food, and other basic necessities were deducted, there was usually little left to save. In many cases, the money participants earned was sent to help support dependents. Other lines of work usually earned less or required initial capital which participants did not have. Some participants reported being part of a savings club, or trying to put aside money themselves, but this was a slow process. For forced labor victims, there was the added complication that in many cases, the trafficker was a family member with whom they lived. This person was their source of housing, meals and all basic necessities. Participants had nowhere else to go, they were frequently underage, and because of their lack of education, had few other employment prospects. One participant explained the hopelessness of her situation saying, *“No, l will stay with my stepmother [exploiter] because she’s the only family I have, however much she mistreats me. If I try to do it, she can call the chairman, people in the village, and also beat me to death”* (Forced labor, Female, age 11).

#### Barriers to seeking help

While being exploited, participants experienced many barriers to seeking help. A major barrier was the lack of awareness of the organizations and resources available to them. Most participants were unable to list any organizations that could help them by name. Among participants that had sought help, the police and healthcare professionals were the most frequently approached. Participants recounted having reported stolen property, rape and in a few select cases, the trafficker, to the police. Physical injuries and STI symptoms were the most common reason medical staff were consulted. However, encounters with these professionals were not always positive experiences. One participant described how he was not believed when he reported sexual assault to the police,

At night, he wanted to sodomize me … While I was asleep, he started to remove my trouser. I ran away and went to the police. When I reached at police, I narrated my story, but they instead called me a mad person. They said that I didn’t wear my trouser properly and my words were false. – Forced labor, Male, age 13

The fact that many help services required money including services like filing a police report, receiving medication, and getting an abortion, acted as a deterrent to seeking help. In some cases, when participants did not have the required money, help services employees exploited participants’ vulnerability and requested sexual favors instead.

By the time you take there your case to him [police officer], and he is aware that you are a sex worker, he doesn’t take you as someone important … he wants to use you. Not once! Not twice! Many have been taken advantage of in the offices. You take there your case and the man says that, "If you want my help, first remove your underwear". Will you refuse? If you refuse, your case won’t be worked on. – Sex trafficking, Female, age 30

Fear also played a strong role in participants’ decision to not seek help. Participants reported feeling threatened by the trafficker who warned them that they or their family would be hurt or killed if they left or talked to anyone about the exploitation. Participants also feared the help services describing that sometimes help services were complicit in the abuse, worked together with the trafficker, or were the clients of their exploitation.

Going to the police … you might want to take there your issues and you find that they know her [exploiter]. So, there is nothing they are going to help with. Instead, you are the one who is considered to be in a mistake when you reach. I had a friend of mine sometime back who went there [the police], but it ended on her being imprisoned. She is the one who was taken to be having a criminal case. Like now the police that is nearby, they know our boss. So, even if went there, would be no help. – Sex trafficking, Female, age 23

Sometimes seeking help was made difficult by restrictions on freedom of movement and communication placed on participants by the trafficker.

Where would you report? There was nowhere to report. They could not even allow moving out of that house. …We were inside there as prisoners. At the gate, there was a security guard and he was commanded that anyone that dared to move out, bullet. There were even two of our friends that were killed when they tried to escape. – Sex trafficking, Female, age 24

Especially in those cases where the trafficker was a family member (mainly for those involved in forced labor) participants’ loyalty and personal ties to the trafficker caused them to be conflicted about seeking help. One participant described this dilemma saying, *“I don’t want the police to arrest my mother, because [participant becomes emotional] I love her so much”* (Forced labor, Female, age 12).

If the trafficker were to be arrested, participants would lose their source of income which caused some participants to be hesitant about seeking help.

Shame and fear of gossip also formed important barriers to seeking help. Participants were weary of confiding in others, and in many cases their family was unaware of the exploitation. A participant explained why she could not open up about her situation saying, “*Some people are not easy. You tell them your problems and they will tell them to anybody else. So it is better to stay quiet and live on.”* (Sex trafficking, Female, age 15).

#### Facilitators of help-seeking

A minority of participants were aware of different non-governmental organizations (NGOs) that offered assistance to victims of human trafficking. These organizations offered services like legal counsel and sometimes vocational training. In those cases where participants sought help, this sometimes had a positive impact. Incidents were described in which seeking help from the police led to the recovery of stolen goods and the arrest of perpetrators. Similarly, seeking medical care contributed to resolution of physical symptoms. Multiple participants also reported receiving help from strangers. Usually, this help was a one-off and consisted of receiving a small amount of money, some food, or assistance with transport. Support was also offered by friends and co-workers. Participants reported appreciating the ability to confide in them and share their experiences. The advice they received from their social network, however, was often not very useful and was usually limited to encouragement to endure.

## Discussion

Interviews with victims of forced labor and sex trafficking in Kampala, Uganda, illustrate the complexity of human trafficking and reveal the substantial physical and psychological morbidity associated with it. Vulnerability to human trafficking can be traced back to financial hardship and abusive home situations in rural villages not uncommonly triggered by the death of a victim’s caretaker. These dire circumstances may cause children to drop out of school and accept work opportunities and promises of a better life that are offered to them. When these opportunities and promises prove false, their limited educational background coupled with the unfamiliar surroundings they find themselves in, where they lack a support system and the basic needs for survival, leave them vulnerable to being trafficked. Their lack of other employment prospects stemming from curtailed education, little to no vocational training, and reliance on income from trafficking for survival, sustain the cycle of exploitation. Victims are generally unaware of organizations that can offer assistance and help is rarely sought out of fear of retribution by the trafficker and other negative consequences. Negative accounts of what happened when participants did seek help, including not being believed, accusations of criminal activities, shaming, and exploitation of their vulnerability, illustrate that this mistrust is not always misplaced.

The victims of human trafficking who participated in this study reported being exploited, exposed to dangerous working conditions, and suffering a host of horrific forms of abuse. Physical abuse and hazardous working conditions led to injuries including lacerations, contusions, and fractures. Forced unsafe sex put forced labor and sex trafficking victims at risk of sexually transmitted diseases, unwanted pregnancy, and the debilitating sequalae of traumatic fistulae. Human trafficking victims in this study were systematically made to feel worthless and depressive symptoms, symptoms of post-traumatic stress disorder, and negative self-perception were common. Our findings are in line with data from other human trafficking studies in Africa which showed psychological, sexual, and physical violence against human trafficking victims at different stages of the trafficking cycle [18–21]. For example, in a mixed-methods study among human trafficking survivors in Nigeria and Uganda, sexual violence and hunger were reported during transit, and abusive working conditions, restricted freedom, and sexual and physical violence were reported during the exploitation phase [18]. A retrospective cohort study in Ethiopia among 671 trafficked women found prevalent sexual violence during predeparture, in transit, and the trafficking destination [19], and among 43 trafficked children in Ghana, maltreatment including starvation, sleep deprivation, denial of healthcare, and verbal and physical abuse was found [21].

The interviews make it clear that much of the vulnerability to trafficking in Uganda is rooted in structural factors like poverty. While the Government of Uganda has made strides in poverty eradication and has succeeded in lowering the proportion of the population living in monetary poverty from 56% in 1992 to 21% in 2017, a United Nations Children's Fund (UNICEF) report revealed that nearly half of Ugandan households (47%) continue to experience multidimensional poverty [22]. Poverty is not uniformly distributed in Uganda with a higher prevalence of multidimensional poverty found in rural (55%) compared to urban (23%) settings, which most of the interview participants originated form [22].

Despite the existence of the Universal Primary Education (UPE) program, under which primary education in Uganda is free, hidden costs associated with uniform purchase, school meals and examination fees are the motivation behind stopping education for six out of ten children who leave school [23]. Our interviews confirm this finding, illustrating how the additional costs associated with school attendance form a barrier to education for children from families facing financial hardship. Research shows that girls in Uganda in particular suffer from financial barriers to education as male children are often prioritized when funds are insufficient [23]. Discontinuation of education is problematic given the strong correlation between education levels and the likelihood of attaining stable employment for young people in Uganda [24]. The financial hardship described in our interviews was often triggered by the death of a caretaker, with almost half of interview participants reporting to have lost a family member. The apparent ubiquitousness of early death in this setting, also noted in other human trafficking research from Uganda and Nigeria, is a worrying public health finding in and of itself and raises questions about the cause of this mortality [18]. The HIV epidemic, armed conflict and natural hazards and disasters likely contribute to these untimely fatalities, once again underlining the need for holistic public health interventions. Research from other African settings shows limited human trafficking awareness, particularly in rural areas and among youth. This lack of awareness among potential victims and others in a position to protect them makes it unlikely that they will question opportunities offered to them, feeding into trafficking vulnerability [25, 26].

We discovered that despite the substantial barriers victims faced to help-seeking, they succeeded in accessing health services for the somatic sequelae of exploitation. Services sought commonly included treatment for acute injuries and illnesses as well as reproductive health services. This highlights the unique position that healthcare workers are in as some of the few professionals that interact with this largely hidden and highly isolated group [27–29]. Health workers have successfully been leveraged in Uganda to improve access to care for other marginalized groups such as men who have sex with men and may be able to play a role in administering human trafficking interventions [30]. As a result of low awareness and gaps in knowledge among health professionals, however, human trafficking is often not recognized and opportunities to identify and help trafficking victims are missed [31–34]. Providers lack training on the issue of human trafficking and do not feel confident in their ability to identify victims [35–37]. Training for healthcare workers needs to be developed and woven into medical curricula or provided as continued medical education to ensure providers possess the skills necessary to identify trafficked persons and provide trauma-informed care [38, 39]. Assessment tools to help providers recognize high-risk patients may be helpful in this regard, but few are validated for healthcare settings, especially in low- and middle-income countries.

Once victims are identified, it is imperative that health workers have the resources necessary to safely and appropriately respond to their complex needs. Health systems should have response plans in place which provide practical clinical guidance to health professionals and connect trafficking victims with available services [40]. More research directed towards contextualized and locally specific solutions needs to be funded to determine the best approaches. Interventions that are offered or assistance programs that survivors are linked to should go further than solely addressing medical complaints. Stakeholders involved in the response to human trafficking have emphasized the importance of offering integrated comprehensive interventions and advocate for a ‘one-stop-shop’ at which survivors of human trafficking can receive all the services they need [41]. In a qualitative needs assessment conducted in Ethiopia among key human trafficking stakeholders including service providers, academics, lawyers, and NGO workers, stakeholders underscored that an array of services including shelter, food, legal support, and vocational training should be provided that meets the complex needs of trafficking survivors [41]. Stakeholders highlighted that trafficking victims themselves often consider financial and employment needs to be of greater priority than their physical and mental complaints [41]. A qualitative analysis of in-depth interviews with 37 human trafficking survivors in Ghana who had completed a 9-month post-trafficking assistance program demonstrated that ensuring access to basic necessities including food, clothing, hygiene and sanitation allowed survivors to focus on developing the vocational skills they would need to start a micro-business after program completion [42]. Human trafficking survivors were found to often lack basic life and survival skills as a result of the young age that they left home. By assigning them roles and responsibilities, the program ensured that survivors were informally provided with critical skills including cooking, caring for a home, decision making, and effective communication [43].

The accounts collected in this study emphasize the public health burden associated with human trafficking and the need for interventions that interrupt the human trafficking trajectory. Possible interventions might be grouped into interventions aimed at decreasing vulnerability in villages pre-trafficking, services to support high-(trafficking)risk individuals surviving on city streets, and programs aimed at identifying those in exploitation to facilitate presentation of an exit strategy to reintegrate back into society. Multi-level interventions are necessary to prevent human trafficking [44].

Based on our study findings, we offer the following recommendations. Interventions are needed at all stages of the human trafficking trajectory (Fig. 1). In Uganda, pre-trafficking vulnerability can be addressed through poverty eradication, supporting families in financial need through covering school fees, strengthening health systems, and raising awareness about human trafficking in rural villages. The transition from recruitment to exploitation can be disrupted by advertising free resources to victims at transits centers and other entry points to the city, outreach activities for at risk-youth on the streets of Kampala, and the creation of drop-in centers. The unique position of healthcare workers can be leveraged to identify human trafficking victims and link them to available services. To achieve this, training in the recognition of human trafficking and the provision of trauma-informed care is needed, as well as the development of comprehensive after-care programs.

Our findings should be considered in the context of the study’s limitations. Snowball sampling was used to obtain the sampling frame that yielded the interview participants. Youth already working with UYDEL were asked to assist in study participant identification which may have led to the recruitment of individuals in their direct social circles, perhaps individuals less isolated than other trafficking victims. For the in-depth qualitative interviews, trafficking victims with severe forms of exploitation based on their survey answers were oversampled and their experience may not be representative of all human trafficking victims. Some of the study participants, mainly those with lived experience of forced labor, were as young as eleven years old at the time of participation. Considering their young age and limited educational experience, a different interviewing technique may have been more effective. Finally, the reported findings represent the experiences of the interview participants and may not be generalizable beyond the study population.

## Conclusions

Human trafficking is an issue of serious global public health concern. In Uganda, structural determinants including poverty and dysfunctional family dynamics force children to drop out of school and drive them into a life of exploitation. Forced labor and sex trafficking victims suffer a host of physical, sexual and psychological abuse during exploitation which results in detrimental short- and long-term health impacts. Fear, lack of awareness, restrictions on movement, and a high degree of dependence on exploiters prevent victims from seeking help. Victims are extremely isolated and healthcare providers and the police are some of the few professionals with whom victims interact, but oftentimes with negative consequences. The improved localized understanding of the human trafficking trajectory in Uganda and its drivers gained through this study can inform the design of interventions to address and prevent human trafficking.

### Supplementary Information

**Additional file 1: Appendix A.** Completed consolidated criteria for reporting qualitative research (COREQ) checklist.

**Additional file 2: Appendix B.** Interview guide questions.

## Data Availability

The interview recordings and transcripts generated and analyzed as part of the current study are not publicly available due the sensitive nature of some of the topics covered and potential for identification of the participants, but will be made available by the corresponding author on reasonable request.

## Abbreviations

AIDS: Acquired immunodeficiency syndrome
CUNY: City University of New York
HIV: Human immunodeficiency virus
NGO: Non-governmental organization
STI: Sexually transmitted infection
UGX: Ugandan shilling
UNICEF: United Nations Children's Fund
UYDEL: Uganda Youth Development Link
