# Supplementary material for: Human trafficking risk factors, health impacts, and opportunities for intervention in Uganda: a qualitative analysis
Source: Glob Health Res Policy. 2023 Dec 11;8:52. doi: 10.1186/s41256-023-00332-z (PMC10712038; doi:10.1186/s41256-023-00332-z)
Supplement: Supplementary file 1 — Additional file 1: Appendix A. Completed consolidated criteria for reporting qualitative research (COREQ) checklist. [file 41256_2023_332_MOESM1_ESM.docx]

**Appendix A. Completed consolidated criteria for reporting qualitative research (COREQ) checklist**

| **No** | **Item** | **Guide questions/description** |  |
| --- | --- | --- | --- |
| 1. | Interviewer/facilitator | Which author/s conducted the interview or focus group? | Interviews were conducted by Uganda Youth Development Link (UYDEL) field teams which were trained by and received assistance from Meredith Dank, the study Principal Investigator. |
| 2. | Credentials | What were the researcher's credentials? *E.g. PhD, MD* | Principal investigator: Meredith Dank, PhD  Interviewers: qualified social workers. |
| 3. | Occupation | What was their occupation at the time of the study? | Interviewers were qualified social workers working for the Uganda Youth Development Link (UYDEL) a National Non-Governmental Organization that was started in 1993 with the aim of enhancing the socio-economic transformation of disadvantaged young people aged 10 -24 years through evidence based interventions that: advocate; create linkages to services and information; and encourage social and livelihood skills development for self-reliance.  UYDEL’s current programs specifically target children and young people (both in and out of school) aged 10-25 years that have been abused and exploited in commercial sex work, victims of trafficking, worst forms of child labor, sexual abuse, physical and psychological abuse and drug and substance abuse, adolescent sexual reproductive health, street children/children without family support systems, teenage adolescent mothers and youth from very low socio-economic background. |
| 4. | Gender | Was the researcher male or female? | The field team consisted of six female interviewers. |
| 5. | Experience and training | What experience or training did the researcher have? | UYDEL has previously conducted research studies focusing on exploitation of children. UYDEL leadership and the field team of interviewers were trained by the study Principal Investigator Meredith Dank on protocols, confidentiality, privacy, and all human subject protection protocols as approved by IRB. Training also consisted of identifying trauma reactions, suicidality, conducting research  with vulnerable populations, and vicarious trauma. |
| 6. | Relationship established | Was a relationship established prior to study commencement? | A relationship was established between study Principal Investigator and UYDEL prior to study commencement. No relationship with participants was established by interviewers prior to study commencement. |
| 7. | Participant knowledge of the interviewer | What did the participants know about the researcher? e*.g. personal goals, reasons for doing the research* | The UYDEL field team did not share personal information with the participants, but did inform them of services provided by UYDEL at the end of the interview if the participant wanted assistance. |
| 8. | Interviewer characteristics | What characteristics were reported about the interviewer/facilitator? e.g. *Bias, assumptions, reasons and interests in the research topic* | Interviewers were female social workers working with UYDEL and spoke English, Luganda, Lusoga and Lunyankole. The lead researchers did not inquire about the reasons, biases and assumptions as to why the interviewers were interested in working on this topic. |
| 9. | Methodological orientation and Theory | What methodological orientation was stated to underpin the study? *e.g. grounded theory, discourse analysis, ethnography, phenomenology, content analysis* | Qualitative interviews were analyzed using a combination of inductive and deductive thematic content analysis guided by an adapted conceptual framework of human trafficking. |
| 10. | Sampling | How were participants selected? *e.g. purposive, convenience, consecutive, snowball* | Interview participants were selected through convenience sampling from a group of participants who had completed a quantitative survey on human trafficking with purposive oversampling of participants who had experienced severe forms of exploitation. |
| 11. | Method of approach | How were participants approached? e*.g. face-to-face, telephone, mail, email* | Participants were approached face-to-face by enumerators at the end of their quantitative survey and invited for participation in a qualitative interview. Interested participants were provided with dates and times that field staff would be on site and available to conduct qualitative interviews to eliminate the need to record participant contact information. |
| 12. | Sample size | How many participants were in the study? | 108 interview participants. |
| 13. | Non-participation | How many people refused to participate or dropped out? Reasons? | This information was not recorded. |
| 14. | Setting of data collection | Where was the data collected? e*.g. home, clinic, workplace* | Data collection took place in private rooms at community based organizations and in private spaces in the communities where the participants were recruited. |
| 15. | Presence of non-participants | Was anyone else present besides the participants and researchers? | No |
| 16. | Description of sample | What are the important characteristics of the sample? *e.g. demographic data, date* | For demographic data see table 1. |
| 17. | Interview guide | Were questions, prompts, guides provided by the authors? Was it pilot tested? | An interview protocol was provided by two of the authors (MD and AH) and it was pilot tested. |
| 18. | Repeat interviews | Were repeat interviews carried out? If yes, how many? | No |
| 19. | Audio/visual recording | Did the research use audio or visual recording to collect the data? | Yes, interviews were audio recorded with consent/assent from the participant. |
| 20. | Field notes | Were field notes made during and/or after the interview or focus group? | No |
| 21. | Duration | What was the duration of the interviews or focus group? | Interviews lasted 40 minutes on average. |
| 22. | Data saturation | Was data saturation discussed? | No |
| 23. | Transcripts returned | Were transcripts returned to participants for comment and/or correction? | No |
