# Supplementary material for: Human trafficking risk factors, health impacts, and opportunities for intervention in Uganda: a qualitative analysis
Source: Glob Health Res Policy. 2023 Dec 11;8:52. doi: 10.1186/s41256-023-00332-z (PMC10712038; doi:10.1186/s41256-023-00332-z)
Supplement: Supplementary file 2 — Additional file 2: Appendix B. Interview guide questions. [file 41256_2023_332_MOESM2_ESM.docx]

**Appendix B. Interview guide questions**

**Demographic Characteristics**

1. How old are you?
2. What’s your gender? (*man, woman, transgender woman, transgender man?)*
3. How many years did you finish in school?
4. When did you last go to school?
5. Where are you from (village, district, etc.)?
6. What is your marital status?
7. Do you have any children? If so, how many?
8. Where do you currently live – I am not asking for the address or neighborhood, just the type of place you live (*e.g. family home, streets, work*)?

*Who else do you live with? I don’t want their names, just to know if it’s family, friends, roommates, other residents, etc.*

*How long have you lived there?*

1. Do you have any siblings? If so, how many?
2. What type of work do your parents do?

**History of Earning Money**

The next questions are about all the different ways you have earned money/food:

1. Please briefly describe all of the ways you have earned money/food in your life.
   1. What are you currently doing?
2. What are the main reasons you started trying different avenues for earning money?
3. What activities has your formal work and other money earning activities entailed?
4. How old were you when you first started doing this type of money earning activity?
5. How did you find out about this way to earn money? *(e.g.: recruiter, village middle-man, friend, neighbor, etc)*
6. Did you migrate to the Kampala area to earn money?
   1. If yes, how did you get to this formal job/money earning activity?
   2. Did anyone accompany you from your village?
   3. Did you or your family have to pay a fee for you to get this formal job/money earning activity?
   4. What were you told you were going to do?
   5. What wage were you promised? Were you promised any other benefits (e.g. housing, time off, education, etc.)?
   6. What happened when you arrived at the site where you were told to work?
7. Did you accept this formal job/money earning activity in order to pay off a loan or advance?
   1. If yes, how much was the loan for?
   2. Who was the loan/advance for?
   3. What was the loan/advance for?
8. Was it your decision to take this formal job/money earning activity?
   1. If yes, why did you decide to take the formal job/money earning activity?
   2. If no, please describe the circumstances under which this decision was made for you.
      1. What would have happened if you refused to take the formal job/money earning activity?

**Current Work/Money Earning Situation**

1. Please describe your current work/money earning activities and working conditions (*Prompts: work hours, number of days per week worked, living conditions, meals provided, breaks provided, activities performed*)
2. How long have you been at this site where you are earning money?
3. How many other people are trying to earn money at the same site?
   1. Please describe the people who are also earning money at the same site as you (*Prompts: age, gender, job duties*)
4. Approximately how much money per week do you make?
   1. What other forms of payment do you receive (e.g. in-kind non cash payment – specify what type, debt repayment, etc.)
5. Do you keep all the money you earn?
   1. If yes, how do you spend the money (if at all)?
   2. If no, describe who gets the money and how much they get.
6. Do you ever feel pressured to do these money making activities even when you do not want to?
7. What would happen if you tried to leave without permission?
8. Has anyone ever left without permission?
   1. If yes, what happened?
9. Have you ever been injured while trying to earn money? If so, please describe what happened.
10. Has anyone at formal work/money earning activity (e.g. employer, co-worker, client) ever asked you to engage in intimate activities?
    1. If yes, how long had you known them before they asked you to do this?
    2. Were you expecting this to be part of the job/money earning activities?
    3. Were you able to refuse? If not, what would happen if you did?
    4. Were you expected to engage in these activities regularly as part of your job/money earning activities or was it just once/a handful of times?
    5. Did someone train you to engage in these activities? If yes, who?
    6. How many clients do you see in a week? Of these clients how many do you engage in intimate activities with?
    7. Who decides what prices are charged?
    8. Where do you go with your clients?

**Experiences of Abuse**

1. Have you ever been abused while at your work/money making activities?
   1. If yes, what kind of abuse did you suffer (e.g. physical, emotional, verbal, psychological, financial)?
   2. Who abused you? (e.g. employer, client, co-worker, etc.)
   3. Have you ever told anyone about the abuse?
      1. If yes, what happened?
      2. If no, why not?
   4. How do you protect yourself (if at all)?
2. Are you able to leave when you want?
3. Are you able to freely communicate with friends, family, etc.?
4. What kinds of abuse do other people in similar situations experience?

**Help-Seeking**

1. Do you know of any organizations you could approach for help if someone was treating you wrongly? Have you ever contacted such an organization? If not, why?
2. Did you ever try to approach the local police while there?
   1. If yes, please describe what happened
   2. If not, why not?

**Last Thoughts**

1. Is there anything else that you would like to tell me or discuss that you believe would be important for this study
